# Hydrogen bonds in crystalline d-alanine: diffraction and spectroscopic evidence for differences between enantiomers

**DOI:** 10.1107/S2052252517015573

**Published:** 2018-01-01

**Authors:** Ezequiel A. Belo, Jose E. M. Pereira, Paulo T. C. Freire, Dimitri N. Argyriou, Juergen Eckert, Heloisa N. Bordallo

**Affiliations:** a Faculdade de Física, Universidade Federal do Pará, Belém, Pará, Brazil; b Departamento de Física, Universidade Federal do Ceará, Fortaleza, Ceará, Brazil; c Niels Bohr Institute, University of Copenhagen, Universitetsparken 5, Copenhagen, 2100, Denmark; d European Spallation Source, 176, SE-221 00 Lund, Sweden; e Department of Chemistry, University of South Florida, 4202 East Fowler Ave, Tampa, FL 33620, USA; f Theoretical Division, Los Alamos National Laboratory, Los Alamos, NM 87545, USA

**Keywords:** chirality, structure analysis, configurational change, density-functional-theory-based methods, phase transitions, intermolecular interactions, properties of solids, hydrogen bonding, materials science

## Abstract

Enantiomeric amino acids have specific physiological functions in complex biological systems. Systematic studies focusing on the solid-state properties of d-amino acids are, however, still limited. To shed light on this field, structural and spectroscopic studies of d-alanine using neutron powder diffraction, polarized Raman scattering and *ab initio* calculations of harmonic vibrational frequencies were carried out. Clear changes in the number of vibrational modes are observed as a function of temperature, which can be directly connected to variations of the N—D bond lengths. These results reveal dissimilarities in the structural properties of d-alanine compared with l-alanine.

## Abstract 2

Neutron powder diffraction from d-alanine, a d-form amino acid present in higher animals including humans, shows subtle structural differences when compared with its enantiomer, l-alanine. These dissimilarities are due to different rearrangements of the NH_3_^+^ group as revealed by Raman scattering.

## 1. Introduction   

It is well known that chirality plays a fundamental role in the bioactivity of molecules taking part in vital functions of living organisms. Amino acids are fundamental molecules of life and can, except for glycine, be found in enantiomeric l- and d-forms with their chiral centres on the α-carbon. Moreover, progress in analytical chemical tools has now established that considerable amounts of d-amino acids are also found in higher mammals including humans (Karakawa *et al.*, 2015 ▸). Their distribution and regulation are different from those of the l-forms. It is well known that d-alanine (d-Ala), d-serine (d-Ser) and d-aspartic acid (d-Asp) can be viewed as the main targets for physiological functions and the diagnosis of various diseases, such as chronic kidney disease, endocrine glands disorders, and schizophrenia (Hamase *et al.*, 2002 ▸).

Although d-amino acids are now increasingly recognized as physiologically active molecules, as well as potential biomarkers (Kimura *et al.*, 2016 ▸), remarkably few studies have been devoted to the understanding of their solid-state properties. As anticipated, most reports do not find any difference in their properties when compared with their chiral counterpart, except in experiments where the chiral character is relevant (Ganesan *et al.*, 2013 ▸; Ishikawa *et al.*, 2017 ▸). Nevertheless, a number of papers from W. Wang’s group (Wang *et al.*, 2000 ▸, 2002 ▸) have described possible phase transitions in single crystals of d-Ala based on observed differences between crystalline d-Ala and l-alanine (hereafter l-Ala) as a function of temperature. These differences were related to the parity violation energy difference (PVED), which has been searched for in chiral molecules since its conjecture by A. Salam in 1992 (Salam, 1992 ▸). However, no evidence for this theory has been obtained to date. For instance, Sullivan *et al.* (2003 ▸) have re-examined these measurements, and as well carried out X-ray diffraction and ^13^C solid-state NMR in both enantiomers of alanine between room temperature and about 250 K, and found no anomalous behaviour. Along these lines Wilson *et al.* (2005 ▸) have further investigated the crystal structures of hydrogenated l-Ala (at 295 and 60 K) and d-Ala (at 300, 295, 260, 250, 240 and 60 K) using single-crystal neutron diffraction. Once more, no clear structural changes were found, which could have supported the anomalies observed in the bulk measurements, and in turn be indicative of the observable effect of PVED.

Careful scrutiny of the results reported in Wilson *et al.* (2005 ▸), however, led us to realise that changes in the N—H covalent bond distances as a function of temperature in d-Ala appeared to be different from those observed for l-Ala. The data for fully hydrogenated single-crystal results from Wilson *et al.* (2005 ▸) are given as supplementary information (Fig. S1). Data obtained by Lehmann *et al.* (1972 ▸) and Destro *et al.* (2008 ▸) for l-Ala are also reported. These findings as well as the biological relevance of d-Ala gave rise to the investigations reported in this paper. Here the structural stability of fully deuterated d-Ala was analysed by means of neutron powder diffraction (NPD), while the dynamics of hydrogenated d-Ala were analysed using single-crystal polarized Raman spectroscopy (RS).

Herein we report on a number of differences in the dynamical behaviour of d-Ala compared with l-Ala, the most remarkable being the observation of new Raman active modes in the *A*- and *B*-irreducible representations of the factor group *D_2_* below 160 and 260 K, respectively. These findings together with the neutron powder diffraction and density-functional-theory-based methods (DFT) results show that small differences in the crystal packing, which were previously disregarded (Wilson *et al.*, 2005 ▸), can indeed induce different rearrangements of the NH_3_^+^ group in d-Ala and l-Ala. We note that the macroscopic symmetry of the crystal is nevertheless preserved.

## 2. Experimental details   

Polarized Raman spectra were collected on hydrogenated C_3_H_7_NO_2_ single crystals obtained by a slow evaporation technique from the preparation of saturated solutions using the solubility curve of d-Ala (Dalton & Schmidt, 1933 ▸). Crystals of a few millimetres in size were obtained by this method, powered again and reutilized for preparation of new saturated solutions. This process was repeated three times in order to obtain crystals with higher quality. The crystals were polished and cut into three parallelepipeds with approximately 90 mm^3^ volume, such that each has *a*, *b* and *c* crystallographic axes perpendicular to the corresponding faces. The 514 nm line of an argon ion laser operating with an output power of 250 mW along with a detection system (Horiba T64000 triple spectrometer) coupled to a liquid nitro­gen cooling charge-coupled-device (CCD) detector was used to collect the data. The samples were mounted in a cryogenic helium closed-cycle system where the temperature could be varied continuously from 295 K to 20 K and maintained constant within ±0.5 K. Six different scattering geometries, 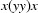, 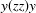, 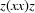, 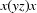, 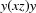, 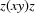, were analysed in the backscattering geometry.

**Figure 1:**
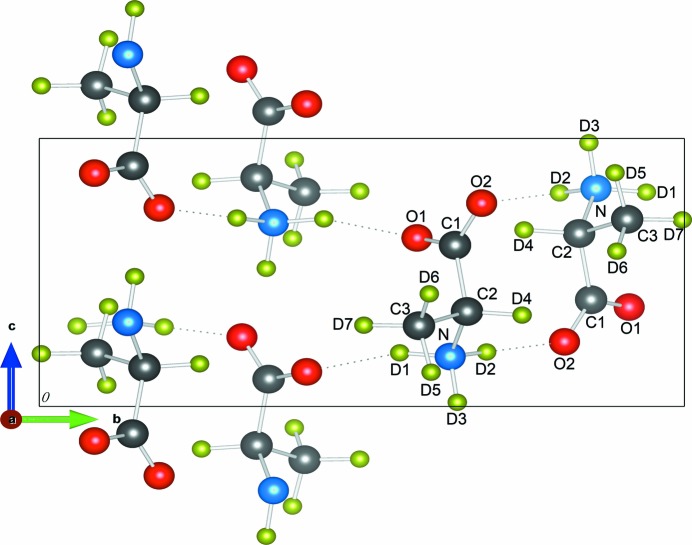
Crystal structure of fully deuterated d-alanine (d-Ala, C_3_D_7_NO) projected onto the *ab* plane with atoms labelled as in Destro *et al.* (1988 ▸). The C atoms are shown in grey, O atoms in red, N in blue and D atoms in green.

Neutron powder diffraction (NPD) measurements on the fully deuterated d-Ala, C_3_D_7_NO_2_, purchased from Cambridge Isotope Laboratories and used without further treatment, were performed on the D2B diffractometer located at the ILL. Data were collected at a wavelength of 1.594 Å in small temperature intervals between 4 and 280 K. This instrument is well suited for an accurate determination of lattice constants, the internal atomic coordinates and a refinement of crystal structure with high resolution. The temperature-dependent powder diffraction data were analysed using the crystallographic model of Destro *et al.* (1988 ▸) as initial input with the *GSAS* suite of programs (Larson & Von Dreele, 1994 ▸). The labelling scheme of the atoms is shown in Fig. 1 ▸. Atomic positions were refined together with lattice constants, isotropic atomic displacement parameters and instrumental peak shape parameters. Special attention was paid to modelling of the background, as the thermal diffuse scattering contribution is significant.

Two types of *ab initio* calculations of harmonic vibrational frequencies were carried out to assist with the analysis of the vibrational spectra. The Raman spectrum was calculated for the isolated molecule using *Gaussian*09 (Frisch *et al.*, 2009 ▸), using as reference the atomic coordinates of the d-Ala at 280 K. The structure was optimized using the polarized continuum model (PCM) of the self-consistent reaction field (SCRF) theory together with the DFT B3LYP level of theory using a 6–311++*G*(*d*,*p*) basis set. Vibrational frequencies of crystalline d-Ala were obtained by periodic calculations on a 2 × 1 × 2 supercell of the alanine crystals with the *Vienna Ab initio Simulation Package* (VASP) (Kresse & Furthmüller, 1996 ▸), using the Perdew–Burke–Ernzerhof (PBE) functional along with Vanderbilt ultrasoft pseudopotentials (Perdew *et al.*, 1996 ▸) with a plane wave kinetic energy cutoff of 450 meV. A 4 × 4 × 4 Monkhorst–Pack mesh of *k* points (Monkhorst & Pack, 1976 ▸) was used to further improve agreement at lower frequencies. This methodology was first applied to optimize the positions of the atoms within the 2 × 1 × 2 supercell. The optimized atomic positions were in turn used to calculate the harmonic frequencies and atomic vibrational amplitudes in the enantiomers for computing the INS spectra, including folding with the experimental resolution function using the program *aClimax* (Ramirez-Cuesta, 2004 ▸).

## 3. Results and discussion   

### 3.1. Raman scattering: anomalies in the lattice modes of hydrogenated d-Ala   

In this section we will discuss the Raman spectra of d-Ala between 30 and 180 cm^−1^ for six different scattering geometries [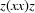, 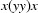, 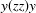, 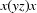, 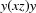, 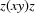] and between 200 and 600 cm^−1^ in the 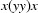, 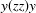 geometries as a function of temperature. Frequency changes in the low spectral region will give insight into deformation of the crystalline lattice, while in the medium region of the spectrum we can follow the evolution of the NH_3_^+^ group, which in l-Ala appears at around 480 cm^−1^ (Susi & Byler, 1980 ▸; Bordallo *et al.*, 1997 ▸; Zhang *et al.*, 2015 ▸).

According to a group theory analysis, the d-Ala crystal possesses 153 optical modes divided into the irreducible representations of *D_2_* factor group as 39 *A* + 38 *B*_1_ + 38 *B*_2_ + 38 *B*_3_ of which 132 modes are internal modes and 21 modes are external, distributed into 12 librations and 9 translations. Modes observed in the Raman spectra of 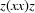, 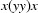, 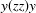 scattering geometries are non-polar representing the Raman tensor components *α_xx_*, *α_yy_* and *α_zz_*. From the theoretical group analysis, six Raman active modes are expected at low wavenumbers, divided into three translational (*T*) and three librational (*L*) modes. *L*-modes, in particular, can be understood as hindered rotations about three perpendicular axes *u*, *v* and *w*, where *u* is nearly parallel to the crystal *c* axis (where a chain of hydrogen bonds links adjacent molecules), *v* is parallel to the long molecular axis and *w* is defined as perpendicular to the plane of the molecule (Loh, 1975 ▸; Crowell & Chronister, 1993 ▸). The two modes of lowest energy, at ~40 and 48 cm^−1^, observed in almost all scattering geometries, are assigned to *w*-axis librations (Loh, 1975 ▸; Crowell & Chronister, 1993 ▸).

**Figure 2:**
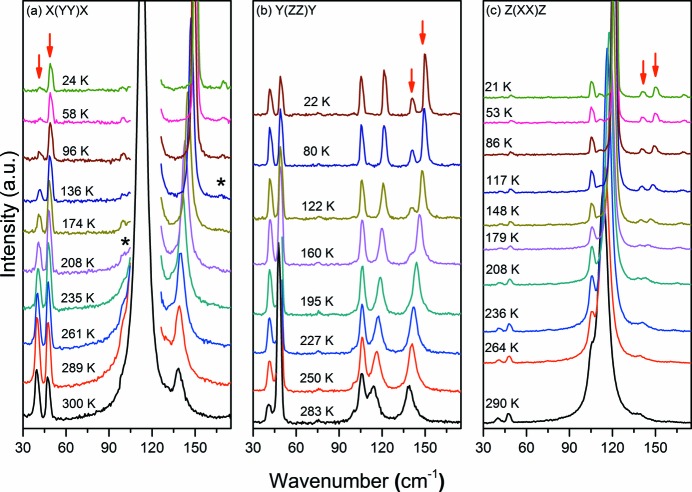
Raman spectra of hydrogenated d-Ala (C_3_H_7_NO_2_) in the *A*-irreducible representation of the factor group *D*_2_ for several temperatures between 20 and 300 K in the region from 30 to 175 cm^−1^. New bands are marked by (*), while arrows indicate bands that split at lower temperature.

**Figure 3:**
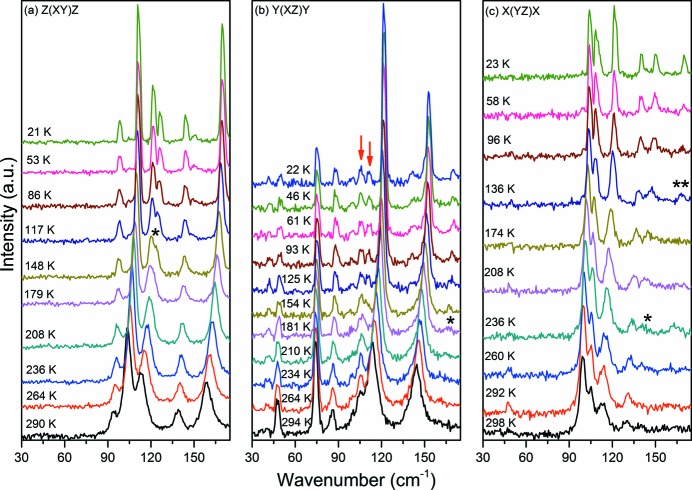
Raman spectra of hydrogenated d-Ala (C_3_H_7_NO_2_) in the *B*-irreducible representation of the factor group *D*_2_ for several temperatures between 20 and 300 K in the region from 30 to 175 cm^−1^. New bands are marked by (*), while arrows indicate bands that split at lower temperature.

Initially, we discuss the temperature-dependent Raman spectra of d-Ala for the 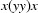 scattering geometry (*A* irreducible representation of the factor group *D*_2_) shown in Fig. 2 ▸(*a*). In this figure it is important to consider the behaviour of the bands at 41 and 48 cm^−1^, indicated by arrows, which present a notable change of intensity at low temperatures. Interestingly, in the spectrum recorded at room temperature the intensity of the band at 41 cm^−1^ is greater than the intensity of the band at 48 cm^−1^, while in the spectrum taken at the lowest temperature, the intensity of the first band is almost zero. It is also possible to observe two small bands, marked by (*), at about 100 and 170 cm^−1^ below 235 and 175 K, respectively. In spite of their very low intensities, the observation of these *L*-modes (Loh, 1975 ▸; Crowell & Chronister, 1993 ▸) shows a trend in which molecules of d-Ala seem to gain librational degrees of freedom. We point out that both modes belong to the *B* symmetry, see Fig. 3 ▸, and a possible explanation for their appearance is a break of the selection rules due to a subtle phase transition or configurational change in d-Ala. Even if one might consider this observation disputable, and that in reality these new bands are already present at 300 K, becoming visible on cooling due to their low intensities, other indictations of a configurational change in d-Ala are clear from the RS results.

Let us first consider Fig. 2 ▸(*b*), the spectra of d-Ala recorded in the 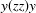 scattering geometry (also *A* irreducible representation). In this spectra we observe at 283 K the presence of a single band located at ~140 cm^−1^, which clearly splits below 160 K in two modes located at 140 and 150 cm^−1^ (marked by arrows) at the lowest temperature. Now we turn to the 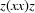 Raman spectra (also an *A* irreducible representation of the factor group *D*_2_) depicted in Fig. 2 ▸(*c*). It is clear that the mode at 140 cm^−1^ also splits into two modes with wavenumbers 140 and 150 cm^−1^ (marked by arrows) at the lowest temperature. Finally, the disappearance of the lowest band at 41 cm^−1^ and the decrease in intensity of the vibration at 48 cm^−1^ on cooling, also seen in Fig. 2 ▸(*a*), further substantiate the idea that crystalline d-Ala undergoes a structural rearrangement at low temperatures.

Now we turn to the polar modes belonging to the *B*-irreducible representations of the factor group *D*_2_, Fig. 3 ▸. Off diagonal Raman tensor components *α_xy_*, *α_xz_* and *α_yz_* were measured in the following scattering geometries:, 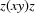, 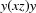 and 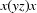. According to a group theory analysis, the modes observed in these configurations are both IR and Raman active. Moreover, due to their scattering geometry, *i.e.* longitudinal optical phonons that generate a macroscopic electric field producing additional scattering mechanism, it is expected that substantial deformation of the crystalline lattice will be distinctly reflected in their behaviour. At 290 K five modes, exactly as predicted, were observed in the 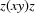 spectrum, Fig. 3 ▸(*a*), at 93, 103, 113, 138 and 159 cm^−1^. However below 180 K the mode at 113 cm^−1^ splits, giving rise to a new peak marked by (*), and at the lowest temperature the modes are observed at 121 and 126 cm^−1^. In Fig. 3 ▸(*b*) we present the 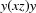 spectrum that shows six modes located at 47, 74, 86, 105, 113 and 144 cm^−1^ at room temperature. Even if in this geometry the signal-to-noise ratio is not top quality, it is possible to observe that the mode located at 105 cm^−1^ starts splitting below 180 K and becomes completely separated, 105 and 110 cm^−1^, at the lowest temperatures (marked by arrows). In addition, an extremely weak mode at 165 cm^−1^, marked by (*), can be observed below 210 K. Finally, in Fig. 3 ▸(*c*) the 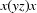 Raman spectrum is presented, showing five modes at 99, 105, 114, 131 and 138 cm^−1^. On cooling two new modes are observed. One is seen around 260 K [marked by (*)] and the other around 136 K [marked by (**)] located at 150 and 169 cm^−1^, respectively, at the lowest temperature. Additionally, an inversion of the intensities of the modes located at 99, 105 and 114 cm^−1^ occurs. The mode at 47 cm^−1^, which completely disappears on cooling, is most likely a leak of polarization due to imperfections on the crystal faces.

**Figure 4:**
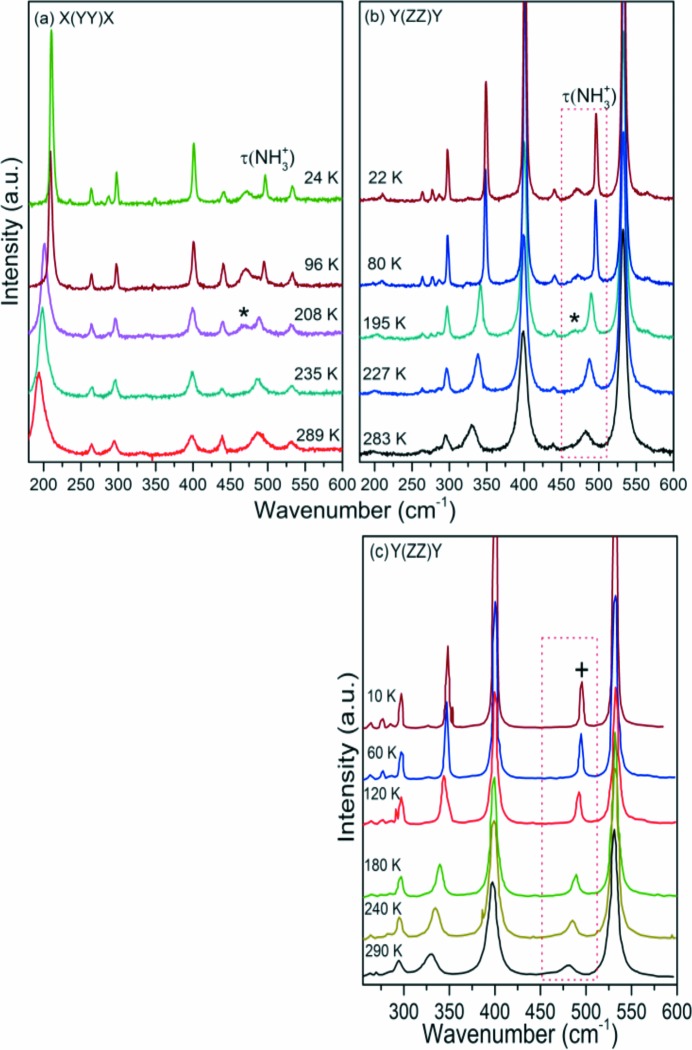
(*a*) and (*b*) Raman spectra of hydrogenated d-Ala (C_3_H_7_NO_2_) in the *A*-irreducible representation of the factor group *D*_2_ for several temperatures between 20 and 300 K in the region from 180 to 600 cm^−1^. A temperature-dependent band at 468 cm^−1^ appears below 200 K and is marked by (*). (*c*) Raman spectra in the *A*-irreducible 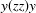 representation of the factor group *D*_2_ for selected temperatures between 10 and 290 K in the region from 250 to 600 cm ^−1^ of hydrogenated l-Ala [C_3_H_7_NO_2_, adapted from (Vik *et al.*, 2005 ▸)]. Note that, differently from d-Ala, the τ(NH_3_^+^) mode indicated for clarity by (*) in (*b*), does not split in l-Ala on cooling, as indicated by (+). However, on heating a remarkable wavenumber decrease accompanied by the increase in the linewidth, attributed to the increase in anharmonicity of the torsional vibrations, is observed in both samples.

**Figure 5:**
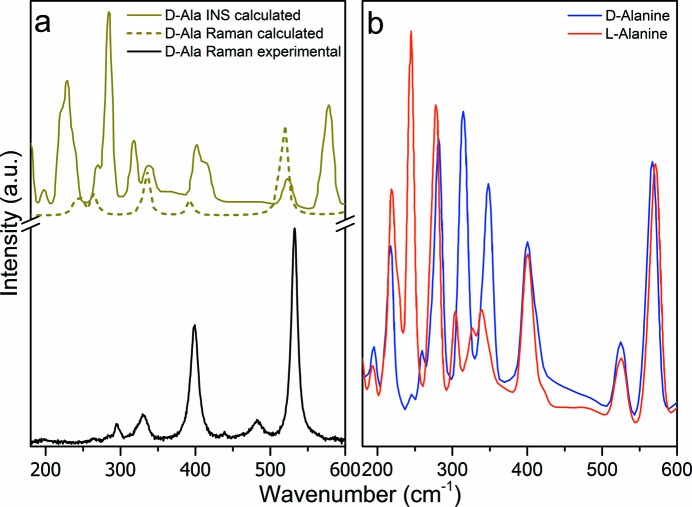
(*a*) Calculated and experimental Raman spectra of d-Ala (C_3_D_7_NO_2_ and C_3_H_7_NO_2_, respectively) between 150 and 600 cm^−1^ at 280 K. Comparison is also made with the calculated INS spectrum at 10 K for d-Ala (C_3_H_7_NO_2_) from the periodic DFT calculations. Here we note that in DFT calculations (0 K) there is no internal mode between 420 cm^−1^ and 500 cm^−1^. (*b*) Comparison of the periodic DFT calculations for fully hydrogenated l- and d-Ala at 10 K where significant differences in the intensities of some of the peaks, *i.e.* the attendant vibrational amplitudes, are evident between about 200 cm^−1^ and about 300 cm^−1^.

In the spectral range between 180 and 600 cm^−1^ (Fig. 4 ▸), we can observe four strong bands at 300, 400, 496 and 532 cm^−1^ (at 22 K) in the 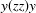 configuration, which are attributed to the CH_3_ torsion, skeletal rocking, NH_3_^+^ torsion (Wang & Storms, 1971 ▸; Barthès *et al.*, 2002 ▸; Kolesov & Boldyreva, 2011 ▸; Zhang *et al.*, 2015 ▸) [labelled in Fig. 4 ▸(*b*) as τ(NH_3_^+^)], and to a mix of intermolecular vibrations. Of more interest, however, is the appearance of a mode at 468 cm^−1^ below 220 K [marked by (*)], distinctively absent in the periodic DFT calculations, which find no mode at all in this region down to 420 cm^−1^ [Fig. 5 ▸(*a*), top lines] as well as in the Raman spectra of l-Ala (Vik *et al.*, 2005 ▸), see Fig. 4 ▸(*c*). This vibration is, however, observed in l-alanine aluminium nitrate, LAAN (Hudson *et al.*, 2009 ▸) at 454 cm^−1^. In LAAN this unassigned vibration has an intensity roughly identical to that of the τ(NH_3_^+^) and is separated by approximately 30 cm^−1^ from the τ(NH_3_^+^) mode (Barthès *et al.*, 2002 ▸; Lagaron, 2002 ▸), therefore its nature was related to an apparent structural change involving motion of a proton at low temperature.

A most noteworthy difference in the low-frequency dynamics of d- and l-alanine is apparent when comparing the calculated 10 K INS spectra for d-Ala *versus* l-Ala, Fig. 5 ▸(*b*). It is quite obvious that there are significant differences in the vibrational amplitudes (*i.e.* peak intensities) of the low-frequency modes below 350 cm^−1^, while the high-frequency portion of the INS spectra for the two crystals are very similar. This result can be considered as a further indication that the intermolecular interactions in l- and d-Ala differ because the local symmetry of the enantiomers is not identical. In the VASP minimized structure, the positions of the atoms not involved in the chirality were found to be the same, while the three hydrogen-bond geometries differ between the enantiomers and were found to be similar to those reported in (Wilson *et al.*, 2005 ▸), see Table S1 in the supplementary information. We can, therefore, hypothesize on the basis of all these observations that in crystalline d-Ala a rearrangement of the hydrogen bonds, and in particular a change in the displacement potential for the NH_3_^+^ protons, may occur which leads to breaking of the selection rules by lowering the local symmetry. In order to better evaluate these spectral anomalies we now turn to the analysis of the neutron powder diffraction data.

### 3.2. Neutron powder diffraction: re-arrangement of the hydrogen bonds in d-Ala   

**Figure 6:**
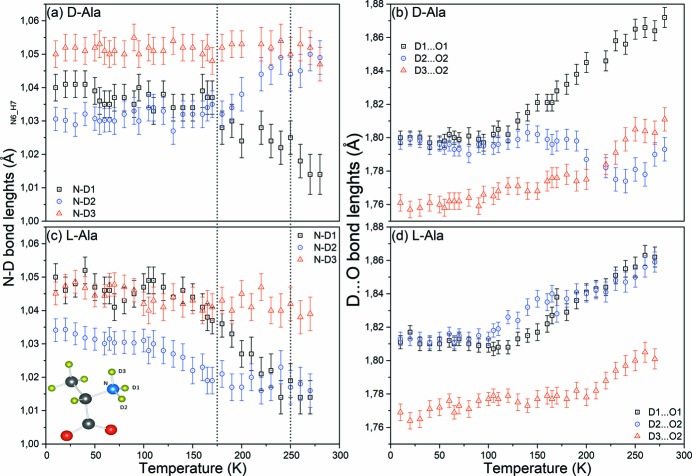
Temperature dependence of the N—D and D···O bond lengths in d-Ala (C_3_D_7_NO_2_) are shown in panels (*a*) and (*b*). Temperature dependence of the N—D and D···O bond lengths in l-Ala reproduced from De Souza *et al.* (2007 ▸) and recalculated from the data used in De Souza *et al.* (2009 ▸), respectively, are shown in panels (*c*) and (*d*). The molecule is represented in the bottom left corner of the figure and labelled in agreement with Destro *et al.* (1988 ▸). The NPD data were collected on D2B (ILL) using λ = 1.594 Å.

Analysis of the NPD data has allowed us to precisely measure the evolution of bond lengths in d- and l-Ala as a function of temperature and draw correlations between their evolution and changes in the Raman data. The advantage of this approach is the self consistency in the data with respect to systematic errors that reveals this evolution of bond lengths as opposed to precise comparisons of bond-length distances to past measurements at a limited number of temperatures made using a variety of instruments and radiations (Lehmann *et al.*, 1972 ▸; Destro *et al.*, 1988 ▸, 2008 ▸; Wilson *et al.*, 2005 ▸). Regardless, our results, Fig. 6 ▸, are in reasonable agreement with the previously reported single-crystal measurements at 300 K on hydrogenated d-Ala (Wilson *et al.*, 2005 ▸), where the NH_3_^+^ group presents two similar N—H distances at this temperature, see Fig. S1(*b*) in the supporting information. We note the agreement of our data with those of Wilson *et al.* (2005 ▸), between 160 and 240 K where also three different N—D distances were observed.

Firstly, turning our attention on the evolution of the N—D bonds as a function of temperature, for both enantiomers, we find a similar behaviour for the N—D1 and N—D3 bonds. While the N—D3 bond, which links the molecules into columns, remains relatively-temperature independent, in contrast we find that the N—D1 bond in both cases has the same value at 280 K and increases on cooling to 175 K, remaining at a relatively constant value below that temperature. The key difference between the N—D bonds of d-Ala and d-Ala resides in the evolution of the N—D2 bond. For d-Ala, we find that this bond length has a similar value to N—D3 at 280 K and on cooling it decreases in value until 175 K, remaining relatively constant in value on further cooling. We find an opposite behaviour in l-Ala, where N—D2 has a similar value to the N—D1 bond length at 270 K, while its value increases gradually on cooling. Overall, the low-temperature behaviour of these bonds in d-Ala and l-Ala is different in that for d-Ala we find that N—D1, N—D2 and N—D3 are dissimilar, while for l-Ala N—D1 and N—D3 are somewhat similar and N—D2 is smaller in value. These data suggest somewhat different conformations in d- and l-Ala both at high and low temperatures. Careful scrutiny of Fig. S1 will lead to this same conclusion.

Turning our attention to the D···O bonds, our measurements also indicate differences in the D···O bond lengths for d-Ala and l-Ala. In both enantiomers, we find the temperature dependence of D(1)···O(1) and D(3)···O(2) to be very similar, both decreasing linearly with temperature. For D(1)···O(1), more specifically, the decrease is linear until approximately 100 K, and then this bond length remains relatively constant with further cooling. The most striking difference in the temperature evolution of these bonds is found for the D(2)···O(2) bond length. For l-Ala, D(2)···O(2) is of similar value and tracks closely the evolution of D(1)···O(1), while in sharp contrast the same D(2)···O(2) bond in d-Ala shows a much smaller value at 280 K compared with its, isomer, increases in size on cooling to 160 K, and on further cooling follows a very similar evolution and value of the D(1)···O(1) bond length.

The dissimilarities in the temperature evolution of bond lengths that we have identified in the NPD data mirror the differences in the low-frequency Raman modes of l- and d-Ala, both reflecting conformational differences between the enantiomers. The differences in the higher temperature behaviour of the D(2)···O(2) and N—D2 bonds, in particular, can be directly correlated with the appearance of the new peaks in the Raman data.

In order to understand these results we turn to previous infrared studies performed on isotopically labelled Nd-Ala molecules in a hydrogenated l-Ala crystal (Rozenberg *et al.*, 2003 ▸), RS studies in fully hydrogenated l-Ala (Kolesov & Boldyreva, 2011 ▸) as well as to more recent studies on the twice methyl­ated amino group of *N*,*N*-di­methyl­glycine (Kapustin *et al.*, 2014 ▸). While Rozenberg *et al.* (2003 ▸) hypothesize that the appearance of the new bands in the spectra of partially deuterated l-Ala reveals an intrinsic hydrogen-bond disorder resulting from different accessible proton positions, the other authors discuss how the N—H···O hydrogen bonds regulate the stability of the main structural unit in crystalline amino acids. Therefore and as a whole, we must consider that while structural methods probe long-range periodic order, RS sensitivity to short-range interactions allows probing heterogeneous hydrogen-bonding systems. Thus, the appearance of the new mode at 468 cm^−1^ and the band splitting of the τ(NH_3_^+^) observed in the RS of d-Ala strongly suggest that the reported structural differences in the two enantiomers are related to dissimilar accessible weakly bounded protons.

## 4. Conclusion   

We have investigated the influence of temperature in the structure of d-Ala combining polarized RS, NPD and DFT-based methods. We find that the reorientation of the NH_3_^+^ group in d-Ala also induces modification of the N—H···O hydrogen bonds between two neighbouring molecules similarly to l-Ala (Vik *et al.*, 2005 ▸). In addition, modes assigned to lattice vibrations (translations and librations of molecules) in the Raman spectrum split on cooling as in l-Ala (Kolesov & Boldyreva, 2011 ▸). What is more interesting and different from l-Ala, is the observation of new Raman active modes in the *A*- and in *B*-irreducible representations for d-Ala below 160 and 260 K, as well as the observation of a temperature-dependent feature at 468 cm^−1^ below 200 K. The temperature dependence of the Raman spectra and the coincidence of the new feature in the Raman spectra with anomalies in the bond lengths obtained from NPD in the deuterated d-Ala exclude the possibility of inclusions in the sample. Finally, from NPD one observes that the temperature dependence of the N—D covalent bonds in d-Ala and l-Ala are quite different in the following way:

(i) For 250 K < *T* < 270 K, the ND_3_^+^ group in l-Ala shows one long (N—D3) and two short (N—D1 and N—D2) covalent bonds, in agreement with Lehmann *et al.* (1972 ▸), while d-Ala has two long (N—D2 and N—D3) and one short (N—D1) covalent bond.

(ii) Between 175 K < *T* < 250 K we observe a transition region for both l- and d-Ala.

(iii) For 60 K < *T* < 175 K, l-Ala shows two long (N—D1 and N—D3) and one short (N—D2) covalent bonds. This is in agreement with Destro *et al.* (2008 ▸) data at 23 K. On the hand, and in agreement with Wilson *et al.* (2005 ▸), d-Ala shows two short (N—D1 and N—D2) and one long (N—D3) covalent bonds.

(iv) Differently from l-Ala, our data suggest the existence of three dissimilar N—D covalent bonds below 60 K in d-Ala. This is further supported by the variation in intensity of the vibration located at 41 cm^−1^ in the 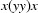 representation; this intense peak observed in the spectrum recorded at room temperature basically vanishes on cooling.

Our work therefore leads to the conclusion that even if the crystal symmetry is maintained both l-Ala and d-Ala undergo micro-conformation transitions due to a subtle rearrangement of the hydrogen-bond network (Barthès *et al.*, 2003 ▸; Kolesov & Boldyreva, 2011 ▸), which is manifested by the evolution of the bond lengths revealed by NPD and the unexpected RS results. Additionally, a slight difference in crystal packing between the two alanine forms induces distinct dynamics for the hydrogen bonds in d-Ala, which culminates in the observation of extra Raman modes and dissimilar hydrogen-bond arrangements compared with l-Ala.

While d-Ala can be used as a biomarker for kidney disease, the presence of d-Ser is now thought to have an important function in the central nervous system, and d-Asp is reported to regulate the hormonal release in the endocrine glands, no comprehensive studies have been conducted to fully understand the solid-state properties of d-amino acids. Therefore, the results presented in this paper could have important clinical implications, since the reported changes in the hydrogen-bond strength of d-Ala when compared with l-Ala, will cause a direct impact on binding energy, consequently affect its affinity, and lead to disequilibrium between active and inactive conformational receptors (Kržan *et al.*, 2016 ▸).

## Supplementary Material

Atomic coordinates for fully deuterated D-alanine obtained using the crystallographic model of Destro et al. (1988) as initial input with the GSAS suite of programs (Larson & Von Dreele, 1994). The data were collected using the neutron powder diffractometer D2B at the ILL.. DOI: 10.1107/S2052252517015573/fs5149sup1.txt

Atomic coordinates for fully deuterated L-alanine obtained using the crystallographic model of Destro et al. (1988) as initial input with the GSAS suite of programs (Larson & Von Dreele, 1994). The data were collected using the neutron powder diffractometer D2B at the ILL.. DOI: 10.1107/S2052252517015573/fs5149sup2.txt

Supporting figures and tables. DOI: 10.1107/S2052252517015573/fs5149sup3.pdf
