# Supplementary material for: Hydrogen bonds in crystalline d-alanine: diffraction and spectroscopic evidence for differences between enantiomers
Source: IUCrJ. 2018 Jan 1;5(Pt 1):6–12. doi: 10.1107/S2052252517015573 (PMC5755572; doi:10.1107/S2052252517015573)
Supplement: Supplementary file 3 [file m-05-00006-sup3.pdf]

IUCrJ

Volume 5 (2018)

Supporting information for article:

Hydrogen bonds in crystalline D-Alanine: Diffraction and spectroscopic evidence for differences between enantiomers

Ezequiel A. Belo, Jose E. M. Pereira, Paulo T. C. Freire, Dimitri N. Argyriou, Juergen Eckert and Heloisa N. Bordallo

Figure S1 summarizes the data reported for fully hydrogenated single crystals results from (Wilson *et al.*, 2005) The lines are meant as guides to the eye. The data at 60 and 295 K were collected using standard procedures on the SXD instrument at the ISIS spallation neutron source at the Rutherford Appleton Laboratory, while the structure refined at 240 K, 250 K, 260 K and 300 K was obtained from data collected on the VIVALDI instrument at the ILL. Data obtained using single crystal X-ray diffraction collected at 23 K (Destro *et al.*, 2008) and single crystal neutron diffration at 300 K (Lehmann *et al.*, 1972) for L-Ala are also reported for comparison.

Figure S2 shows a typical Rietveld analysis of neutron diffraction data measured from D-Ala using the neutron powder diffractometer D2B.

The three N-H bond geometries obtained for L-Ala and D-Ala after performing the VASP minimization of the crystalline structure are given in Table S1 together with the values reported in (Wilson *et al.*, 2005).

Lattice coordinates for D-Ala and L-Ala as a function of temperature obtained from the powder diffraction data analysed using the crystallographic model of Destro *et al.* (1988) as initial input with the GSAS suite of programs (Larson & Von Dreele, 1994) are also provided.

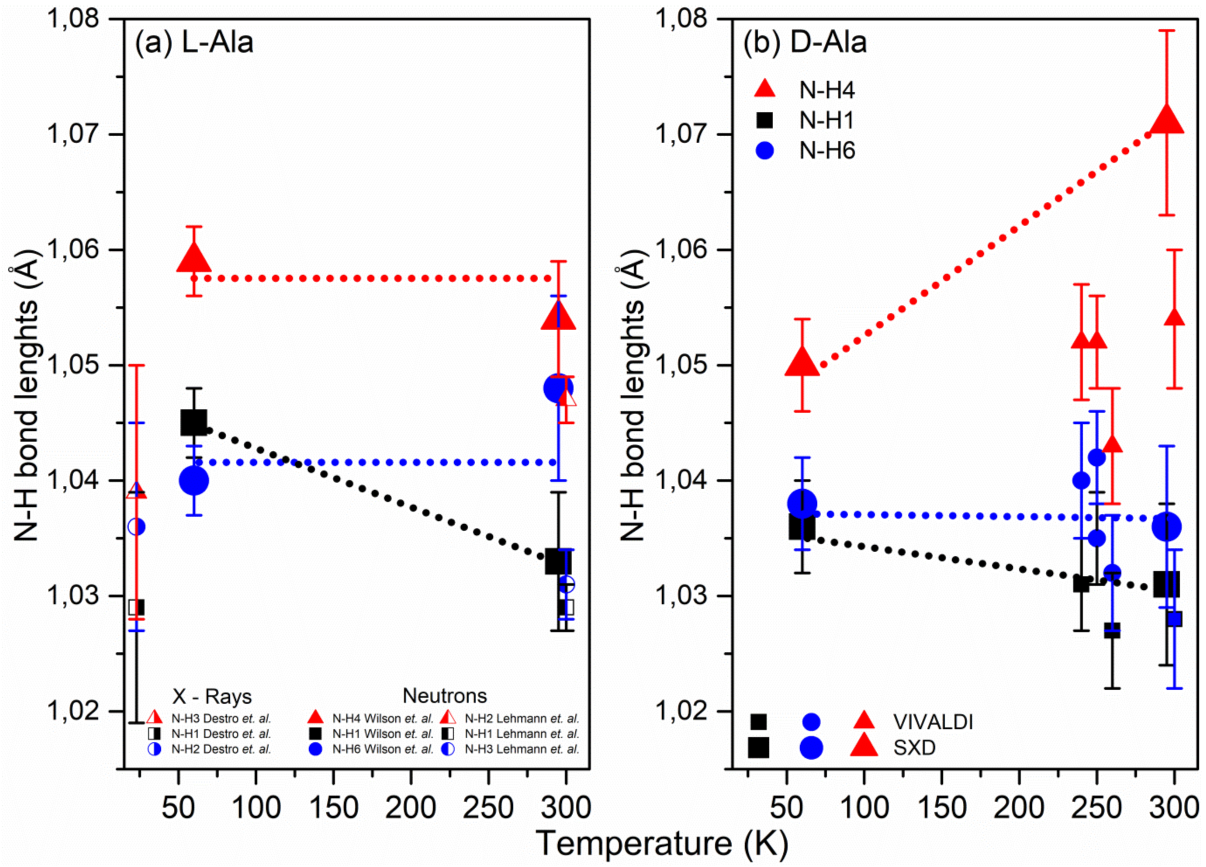

Figure S1 (a) Single crystal X-ray diffraction data reported by Destro *et al.* (2008) (right side half filled symbols) at 23K along with single crystal neutron diffraction data reported by Wilson *et al.* (2005) (closed symbols, data taken using SXD) at 295 and 60K and by Lehmann *et al.* (1972) (left side half filled symbols) at 300K for fully hydrogenated L-Ala. (b) Single crystal neutron diffraction data reported by Wilson *et al.* (2005) at various temperatures using VIVALDI (small closed symbols) and SXD (large closed symbols) for fully hydrogenated D-Ala. On this figure we have kept the bond length nomenclature used by the respective authors to facilitate comparison. Note that we have used the Destro *et al.* (1988 & 2008) nomenclature for labelling the bond lengths of deuterated L-Ala and D-Ala. Thus, square, circle and triangle symbols always correspond N-H_1, N-H_2 and N-H_3, respectively. The lines are meant as guides to the eye.

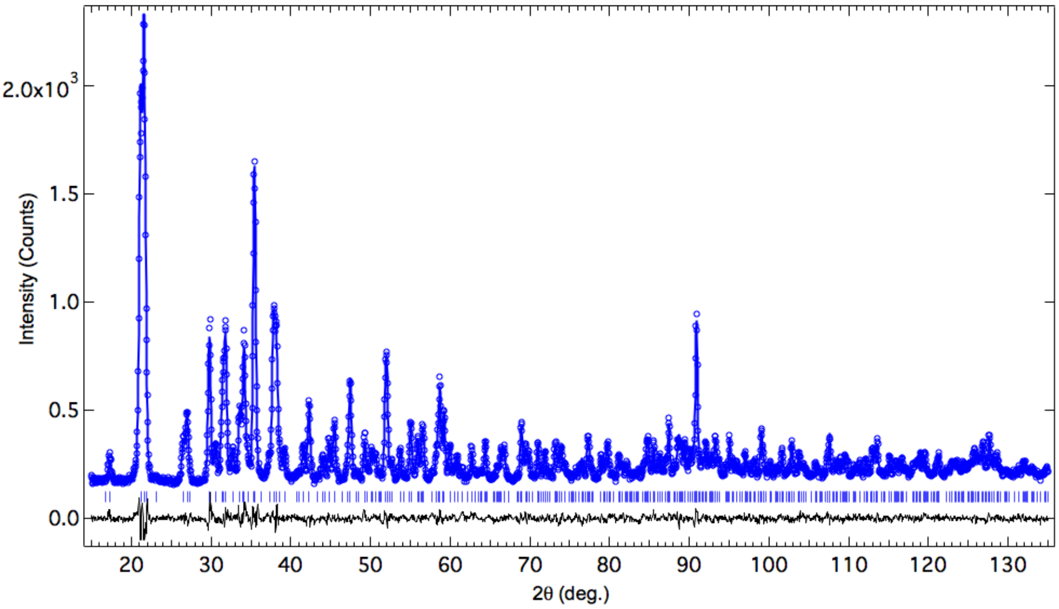

Figure S2 A typical Rietveld analysis of neutron diffraction data measured from D-Ala using the D2B powder diffractometer at the ILL. These data were collected at 270 K. Here the open circles represent the observations and the line through the data points the calculated profile. The difference (ons-calc) is plotted below the data together with the calculated position of Bragg reflections represented as vertical bars.

Table S1 N-H bond distances minimized in the DFT calculations compared to experimental values (Wilson *et al.*, 2005).

N-H distance (Å)	D-Ala DFT minimized structure	D-Ala at 60K Wilson *et al.*, 2005	L-Ala DFT minimized structure	L-Ala at 60K Wilson *et al.*, 2005
$N\text{-}H_1$	1.0434	1.036(4)	1.046	1.045(3)
$N\text{-}H_2$	1.0459	1.038(4)	1.0435	1.040(3)
$N\text{-}H_3$	1.0610	1.050(4)	1.0605	1.059(3)
